# Sleep Duration and Breast Cancer Phenotype

**DOI:** 10.1155/2013/467927

**Published:** 2013-11-04

**Authors:** Ali Khawaja, Santosh Rao, Li Li, Cheryl L. Thompson

**Affiliations:** ^1^Medical College, The Aga Khan University Hospital, Karachi 74800, Pakistan; ^2^Division of Hematology and Oncology, University Hospitals Case Medical Center, 11000 Euclid Avenue, Cleveland, OH 44106, USA; ^3^Department of Family Medicine and Community Health, Case Western Reserve University, 10900 Euclid Avenue, Cleveland, OH 44106, USA; ^4^Department of Epidemiology and Biostatistics, Case Western Reserve University, 10900 Euclid Avenue, Cleveland, OH 44106, USA; ^5^Case Comprehensive Cancer Center, Case Western Reserve University, 10900 Euclid Avenue, Cleveland, OH 44106, USA

## Abstract

Emerging evidence suggests that short sleep is associated with an increased risk of cancer; however, little has been done to study the role of sleep on tumor characteristics. In this study, we evaluated the relationship between sleep duration and tumor phenotype in 972 breast cancer patients. Sleep duration was inversely associated with tumor grade (univariate *P* = 0.032), particularly in postmenopausal women (univariate *P* = 0.018). This association did not reach statistical significance after adjustments for age, race, body mass index, hormone replacement therapy use, alcohol consumption, smoking, and physical activity in the entire study sample (*P* = 0.052), but it remained statistically significant (*P* = 0.049) among post-menopausal patients. We did not observe a statistically significant association between sleep duration and stage at diagnosis, ER, or HER2 receptor status. These results present a modest association between short duration of sleep and higher grade breast cancer in post-menopausal women. Further work needs to be done to validate these findings.

## 1. Introduction

Breast cancer is the leading cancer in women in the United States and the third leading cause of cancer deaths among women [1]. It was estimated that over 220,000 women will be diagnosed in 2012 with breast cancer, with almost 40,000 women deaths [2]. Women diagnosed with localized breast cancer have a very favorable outcome, with a 99% 5-year survival. However, the survival rate decreases to 84% with regional disease and 23% for women with distant metastases, underscoring the importance of identifying factors associated with the development of more aggressive breast cancers.

There is an emerging evidence for the role of sleep deprivation in carcinogenesis, with new research suggesting that disruptions in the circadian rhythm may increase the risk of several types of cancer [3–5], including breast cancer (reviewed in [6]). In the few studies of sleep duration and risk of breast cancer, the association of short sleep duration and the incidence of breast cancer has been mixed, with one study suggesting a decreased risk of breast cancer in women who slept longer [7], two other studies showing an inverse association between sleep duration and risk of breast cancer [8, 9], and a fourth study that did not find evidence of this association [10].

However, much less work has been done to investigate whether short sleep duration prior to the diagnosis is associated with breast cancer phenotype. Markers of more aggressive and advanced breast cancer include stage, determined by the size of the tumor and lymph node status or distant metastases, tumor grade, hormone receptor status, and HER2 expression status. Patients with HER2 positive and/or estrogen receptor (ER) negative breast cancer carry a poorer prognosis with a higher risk of recurrence [11]. For patients with hormone receptor-positive early breast cancer (stage I or II), Oncotype DX, a gene-expression-based marker of increased likelihood of cancer recurrence, has been used to help guide the clinical decisions regarding the benefit of chemotherapy [12]. Oncotype DX recurrence scores correlate with higher tumor grade and lower estrogen receptor expression [13, 14]. We recently reported the association of short sleep duration with higher Oncotype DX recurrence scores among post-menopausal breast cancer patients [15]. 

The aim of this study was to further explore the relationship between sleep deprivation and markers of cancer aggressiveness in breast cancer patients. We examined the association of sleep duration with the stage and grade of tumor as well as tumor markers (ER and HER2 expression). We further stratify by menopausal status, as we hypothesize that the sleep duration would have a disproportionate effect on post-menopausal breast cancer patients, in light of our earlier findings [15].

## 2. Materials and Methods

### 2.1. Patient Population

The longitudinal changes in mammographic density and risk of breast cancer study is an ongoing breast cancer case-control study of women with multiple screening mammograms. Women with newly diagnosed breast cancer (including DCIS) at the University Hospitals Case Medical Center (UHCMC), as well as controls recruited at the time of screening mammography, were approached for recruitment for study participation from January 2007 through July 2012. Exclusion criteria included prior nonsurgical treatment for any cancer, other concurrent cancers, or known presence of BRCA1 or BRCA2 mutation. Patients must speak English to be eligible for participation. Only the breast cancer patients were included in this study. All participants completed a phone survey based on a validated questionnaire developed and used by the researchers at the University of Southern California for breast cancer research [16]. Participants also donated a blood sample for genetic and biomarker studies and consented to link their study data to their medical records. All patients provided informed consent linking their responses to their medical record data, and the study was approved by the UHCMC institutional review board.

### 2.2. Data Collection

Patients enrolled in the study were asked by phone survey about their average length of sleep at night for the 2 years prior to the diagnosis as well as additional lifestyle and demographic factors. Body mass index (BMI) was calculated from self-reported height and weight prior to diagnosis. Physical activity was coded as the sum of the self-reported average weekly hours of strenuous and moderately strenuous physical activity. Smoking was defined as current smoker versus noncurrent smoker, which includes both never smokers and past smokers. Alcohol consumption was based on questions about the average weekly number of drinks of beer or ciders, wine and hard liquor, or mixed drinks in the two years prior to diagnosis. Current drinkers were coded as those individuals reporting drinking at least one drink per week. Menopausal status was defined as post-menopausal for women self-reporting having no menstrual periods within the previous 6 months, including women who have not had a menstrual period due to surgery, and premenopausal for those reporting a recent menstrual period. Race was self-reported as Caucasian, African-American, Hispanic, Asian, or Other. Due to small numbers, Hispanic and Asian participants were coded as “Other.” Current use of hormone replacement therapy (HRT) was defined as self-reported use of estrogen and/or progesterone replacement therapy for one month or longer any time during the two years prior to diagnosis (cases) or two years prior to recruitment (controls). 

Medical records were obtained and reviewed for tumor stage at diagnosis, modified Bloom-Richardson grade, and hormone receptor and HER2 status (when available). When there were concurrent tumors present, the most advanced one was coded. 

### 2.3. Statistical Analysis

Sleep was treated as a continuous variable and categorized into less than 6 hours per night, at least 6 but less than 7 hours per night, and at least 7 hours per night, as we previously found it to be associated with Oncotype DX score (15). The statistical significance of differences in demographic and clinical characteristics among the women in the three sleep categories was done using a chi-square test (for categorical variables), or a one-way analysis of variance (ANOVA) (for continuous variables). Mean hours of sleep per night was calculated for each tumor characteristic, and the statistical significance of differences in means was calculated using ANOVA. In order to assess the univariate trend of increasing stage and grade with hours of sleep, we used an ordinal logistic regression with sleep duration as a continuous variable. 

Average sleep duration is known to vary with age, obesity, and other lifestyle variables. In order to adjust for these potential confounders, we tested the association of self-reported average hours of sleep per night with grade, stage, ER status, and HER2 status using logistic regression models (ordinal logistic regression for grade and stage). In each regression model, we adjusted for age, race, HRT, BMI, current smoking, current alcohol consumption, and physical activity, as all these factors are known to be associated with sleep duration. 

In light of our earlier findings that the association of Oncotype DX score and sleep duration was limited to post-menopausal patients, and the known difference in etiologies of pre- and post-menopausal breast cancer, we then repeated all statistical analyses stratified by menopausal status. All statistics were performed using SAS v.9.2, using *P* values < 0.05 for significance. 

## 3. Results

From a total of 1,097 breast cancer patients participating in the study, we were unable to locate medical records for 118 patients. Two patients were excluded from this analysis due to diagnoses of Paget's disease or Phyllodes tumor. Five additional patients with missing sleep duration data were excluded. The remaining 972 patients were included in the study. 

Table 1 shows the characteristics of the study population overall and by category of sleep duration. There was a significant difference in sleep duration by race, as African American women reported less sleep than Caucasian women (*P* < 0.0001). As expected, women who reported fewer hours of sleep had higher BMI (*P* = 0.015). We also noted that short sleepers were less likely to be current alcohol drinkers (*P* < 0.0001). No clear trend was observed for menopausal status, stage, grade, estrogen receptor status, or HER2 status with sleep duration category. 

Table 2 compares the differences in mean self-reported average hours of sleep per night with different measures of an aggressive breast cancer phenotype. In univariate analyses, a significant inverse relationship was found between sleep category duration and grade, with women with higher grade tumors reporting less sleep prior to diagnosis (*P* = 0.032, Table 2), as well as sleep as a continuous variable and tumor grade (*P* = 0.011). This association was found in post-menopausal women (sleep categories *P* = 0.018, sleep hours as continuous *P* = 0.010), but no trend was observed in pre-menopausal women (sleep categories *P* = 0.022, sleep hours as continuous *P* = 0.59). There was no univariate correlation between hours of sleep per night and stage (*P* = 0.22). Although, as hypothesized, women with positive HER2 status reported fewer hours of sleep on average, it was not statistically significant (ANOVA *P* = 0.17). 

In multivariate analyses adjusting for age, race, BMI, physical activity, current HRT use, tobacco, and alcohol use, average hours of sleep per night no longer remained statistically significantly associated with grade (*P* = 0.052). However, the statistical significance of this association among post-menopausal breast cancer patients remained (*P* = 0.049). As expected, after adjustment, no association among premenopausal breast cancer patients was observed (*P* = 0.46). 

## 4. Discussion

According to the National Sleep Foundation (NSF), a healthy adult should sleep 7–9 hours/day [17]. A survey conducted by the National Health Interview Survey (NHIS) revealed that about 30% of employed US civilian adults report an average sleep duration of ≤6 hours a day, which highlights lack of sufficient sleep as a public health problem [18]. Previous studies suggest that sleep deprivation is a risk factor for the development of breast cancer [8, 9]. We recently showed that short sleep duration correlates with higher Oncotype DX recurrence scores in post-menopausal breast cancer patients [15]. In this study, we show a modest association between short sleep duration and higher grade of breast cancer, which is limited to post-menopausal women. 

Multiple studies have reported an association between short sleep duration and the risk of developing cancer. The biologic basis for this association has yet to be determined. The metabolic syndrome describes the clustering of hypertension, dyslipidemia, obesity, and insulin-resistance [19]. Sleep deprivation is linked with obesity and the metabolic syndrome [20, 21], both of which contribute to breast cancer incidence and stage at presentation, although our multivariate analysis accounted for BMI differences. Turek et al. reported in their animal trial that mice homozygous for a mutant *Clock* gene, a gene that influences both the persistence and periods of circadian rhythms, tend to have a higher food intake, were obese and more prone to develop metabolic syndrome with high levels of leptin, lipids, glucose and reduced compensatory insulin production [22]. Insulin resistance has been shown to be a risk factor for the incidence of breast cancer, again primarily in post-menopausal women, and negatively impacts prognosis [23, 24]. Although insulin resistance is correlated with BMI, we did not specifically adjust for independent effects of insulin resistance. Sleep deprivation also has a proinflammatory and immuno-modulatory effect, which may contribute to the development of more aggressive cancers [25].

It has been observed that genes regulating the sleep cycle are deregulated in cancers [26]. Two sleep-related genes, PERIOD1 and PERIOD3, which are key regulators of our circadian rhythm, have been found to affect the apoptosis in cancer cell lines [27]. A recent study, which used a combination of human breast tumor analysis and mouse models, further showed that a deletion/reduced expression of PERIOD3 gene on chromosome 1p36 was related to the recurrence of the tumor in ER + breast cancer patients, further highlighting the role of the circadian rhythm in tumor aggressiveness [28]. 

Another possible explanation may be found in the role of melatonin in the regulation of the circadian rhythm and its potential effects on the breast cancer. Melatonin depletion has been seen in breast cancer patients, with low nighttime peaks [29], rather than low daily levels [30], correlating with advanced stage at presentation. Antineoplastic properties of melatonin in preclinical models include antioxidant, proapoptotic, antiinflammatory, antiangiogenic, immunostimulatory effects, and alterations in metabolism and gene regulation [31]. It is now well known that the constant exposure to light at night promotes tumorigenesis by inhibiting nocturnal release of melatonin by the pineal gland. It was initially highlighted in an experimental model where either the surgical removal of pineal gland or constant light promoted mammary tumorigenesis in rodents [32]. These findings were further strengthened by the work of Blask et al. [33]. In this study, blood samples were taken from healthy, premenopausal women either in daytime, nighttime, or nighttime after the exposure to bright, white fluorescent light. These blood samples were then perfused in the human breast cancer xenografts in nude rats. It was noted that the nighttime samples with physiological melatonin-rich levels significantly suppressed the tumor proliferative activity and linoleic acid metabolism. However, the melatonin deficient samples following ocular light exposure simulated the increased tumor proliferative activity of the daytime samples. Moreover, this relationship was found to be intensity dependent [33]. In addition, using a tissue isolated human breast xenograft tumor nude rat model, Mao et al. highlighted the link between melatonin and glycogen synthase kinase 3*β* (GSK3*β*), an enzyme that plays a vital role in metabolism and cell proliferation. Melatonin is responsible for activating GSK3*β*, which in turn inhibits epithelial-to-mesenchymal transition, a critical process in underlying cancer spread and metastasis. The exposure to light at night was found to suppress the nocturnal pineal gland melatonin synthesis leading to the inhibition of circadian phosphorylation of GSK3*β* and hence increasing the susceptibility to breast cancer spread [34]. Furthermore, melatonin has multiple antiestrogenic effects, acting to decrease ovarian estrogen production, reducing aromatase activity, and decreasing ER-*α* expression and estrogen mediated transcription of proliferative genes [35]. 

Due predominantly to the large number of DCIS (stage 0) patients in our sample, for which HER2 is not routinely tested, HER2 status was not available for a large proportion of our study population (24.5%). In our sample, patients with HER2 positive breast cancer reported less average nightly sleep. Although this association was not statistically significant, we feel it is worth following up in a larger sample population. There was no clear association between sleep duration and the development of metastatic breast cancer, as premenopausal patients with stage IV disease slept less, whereas post-menopausal patients with stage IV disease slept more, although our sample size in this group was very small.

There are additional limitations in our study. First, the self-reported duration of sleep is subject to recall bias and does not measure other parameters of sleep, including quality and sleep patterns, which may also be important risk factors for breast cancer aggressiveness, but we are unable to study in this sample. However, given that we recruited patients at diagnosis, we feel that this recall bias would be minimal. In addition, we only queried on sleep duration for the two years prior to diagnosis. We cannot address possible effects of sleep earlier in life or the longer term effects of short sleep duration. Secondly, although our study population reflects the racial distribution of the patients seen at this hospital, a majority of our study population is Caucasian. Further work will need to be done in other populations with more minorities to assess generalizability of our findings. A large study population added to the strength of the study; however, the small number of premenopausal patients limits our ability to make conclusions in that subset of patients. However, the observed effect of the association of sleep with grade in the premenopausal population is much smaller than that in the post-menopausal group. Therefore, while we cannot rule out an effect of sleep duration with grade in premenopausal women, our data suggests that if such an effect exists, it is likely to be a smaller effect than in post-menopausal breast cancer patients. 

To the best of our knowledge, this is only the second study to investigate the association between sleep duration and breast cancer phenotype, the first being our study on sleep duration and Oncotype DX score. Approximately 1 in 8 US women will develop breast cancer in their lifetime. With the additional mortality and costs associated with treating more aggressive breast cancers, it is important to understand that risk factors that affect breast cancer severity. Given that 30% of adults sleep less than 6 hours per night [18], the association of short sleep duration with breast cancer incidence and phenotype suggests that the attributable population risk due to short sleep duration is high. Further studies are required to validate these findings, as well as to identify the biological reasons for this observed association. 

## Figures and Tables

**Table 1:** Patient characteristics by category of average hours of sleep.

	All (*N* = 972)	Less than 6 hours of sleep/night (*N* = 105)	6 to less than 7 hours of sleep/night (*N* = 223)	7 or more hours of sleep per night (*N* = 644)	P*
Age, mean (SD)	58.0 (11.4)	57.8 (11.0)	57.3 (11.1)	58.4 (11.6)	0.44
Postmenopausal, *N* (%)					0.87
Yes	817 (84.1%)	88 (10.8%)	190 (23.3%)	539 (66.0%)	
No	155 (16.0%)	17 (11.0%)	33 (21.3%)	105 (67.7%)	
Race, *N* (%)					<0.0001
Caucasian	831 (85.5%)	74 (70.5%)	183 (82.1%)	574 (89.1%)	
African American	126 (13.0%)	28 (26.7%)	37 (16.6%)	61 (9.5%)	
Other	15 (1.5%)	3 (2.9%)	3 (1.4%)	9 (1.4%)	
Body mass index, kg/m^2^, mean (SD)	28.1 (6.5)	29.4 (7.1)	28.7 (7.3)	27.7 (6.1)	0.015
Physical activity per week, hours, mean (SD)	4.0 (4.1)	3.6 (4.6)	4.1 (4.3)	4.0 (3.9)	0.55
Current alcohol use, *N* (%)					<0.0001
Yes	473 (48.7%)	30 (71.4%)	91 (40.8%)	352 (54.7%)	
No	499 (51.3%)	75 (28.6%)	132 (59.2%)	292 (45.3%)	
Current HRT user, *N* (%)					0.63
Yes	143 (14.7%)	16 (15.2%)	37 (16.6%)	90 (14.0%)	
No	829 (85.3%)	89 (84.8%)	186 (83.4%)	554 (86.0%)	
Stage, *N* (%)					0.30
0 (DCIS)	199 (20.5%)	24 (22.9%)	46 (20.6%)	129 (20.0%)	
1	395 (40.6%)	37 (35.2%)	79 (35.4%)	279 (43.3%)	
2	278 (28.6%)	31 (29.5%)	68 (30.5%)	179 (27.8%)	
3	62 (6.4%)	8 (7.6%)	21 (9.4%)	33 (5.1%)	
4	21 (2.2%)	3 (2.9%)	5 (2.2%)	13 (2.0%)	
Missing	17 (1.7%)	2 (1.9%)	4 (1.8%)	11 (1.7%)	
Grade, *N* (%)					0.40
1	145 (14.9%)	10 (9.5%)	30 (13.5%)	105 (16.3%)	
2	309 (31.8%)	35 (33.3%)	68 (30.4%)	206 (32.0%)	
3	209 (21.5%)	23 (21.9%)	53 (23.8%)	133 (20.7%)	
Missing	309 (31.8%)	37 (35.2%)	72 (32.3%)	200 (31.1%)	
ER status					0.0064
Positive	734 (75.5%)	83 (79.0%)	155 (69.5%)	496 (77.0%)	
Negative	173 (17.8%)	15 (14.3%)	56 (25.1%)	105 (16.3%)	
Unknown/not done/missing	65 (6.7%)	7 (6.7%)	12 (5.4%)	46 (7.1%)	
HER2 status					0.38
Positive	135 (13.9%)	17 (16.2%)	34 (15.2%)	84 (13.0%)	
Negative	599 (61.6%)	57 (54.3%)	135 (60.5%)	407 (63.2%)	
Unknown/not done/missing	238 (24.5%)	31 (29.5%)	54 (24.2%)	153 (23.8%)	

*P value of ANOVA (for continuous variables) or chi-square (for categorical variables) test; HRT: hormone replacement therapy.

**Table 2:** Mean self-reported average hours of sleep per night by patient characteristic.

	All patients			Premenopausal			Postmenopausal		
	Hours of sleep per night, mean (SD)	P*	P**	Hours of sleep per night, mean (SD)	P*	P**	Hours of sleep per night, mean (SD)	P*	P**
All	7.03 (1.20)	N/A	N/A	7.08 (1.24)	N/A	N/A	7.02 (1.20)	N/A	N/A
Stage		0.031	0.36		0.40	0.60		0.026	0.26
0 (DCIS)	6.98 (1.20)			7.11 (0.85)			6.95 (1.28)		
1	7.14 (1.20)			7.07 (1.42)			7.15 (1.16)		
2	6.99 (1.16)			7.23 (1.28)			6.94 (1.13)		
3	6.65 (1.14)			6.42 (1.17)			6.67 (1.14)		
4	7.12 (1.68)			6.00 (1.41)			7.24 (1.69)		
Grade		0.032	0.052		0.022	0.89		0.018	0.049
1	7.23 (1.20)			7.63 (1.34)			7.16 (1.17)		
2	7.05 (1.24)			6.75 (1.27)			7.11 (1.23)		
3	6.89 (1.18)			7.27 (1.38)			6.82 (1.13)		
ER status		0.31	0.71		0.38	0.88		0.47	0.69
Positive	7.04 (1.21)			7.10 (1.30)			7.03 (1.19)		
Negative	6.94 (1.19)			6.88 (0.98)			6.95 (1.23)		
HER2 status		0.17	0.23		0.79	0.64		0.17	0.31
Positive	6.91 (1.10)			7.00 (0.89)			6.89 (1.13)		
Negative	7.06 (1.21)			7.09 (1.43)			7.06 (1.18)		

*P value of ANOVA (univariate); **P value of sleep duration in ordinal or standard logistic regression adjusted for age, race, hormone replacement therapy (HRT), family history of breast cancer, body mass index (BMI), current smoking, current alcohol consumption, and physical activity.
